# Management, Practice, and Outcomes of Fragility Fractures and Osteoporosis Care at a Tertiary Hospital in Riyadh, Saudi Arabia

**DOI:** 10.7759/cureus.116907

**Published:** 2026-09-25

**Authors:** Yazeed Alsulaiman, Abdulrahman Alsahli, Abdullah Alamoudi, Rayan Almutairi, Leen Almojel, Dana Alturaifi

**Affiliations:** 1 College of Medicine, King Saud bin Abdulaziz University for Health Sciences, Riyadh, SAU; 2 Family Medicine and Primary Care, King Abdulaziz Medical City, Riyadh, SAU; 3 Internal Medicine, King Abdulaziz Medical City, Riyadh, SAU

**Keywords:** electronic health records, fragility fractures, frailty index, orthopedic outcomes, osteoporosis care gap

## Abstract

Introduction and objectives

Fragility fractures are sentinel events for osteoporosis, yet secondary prevention remains underused, and chronological age alone may not capture the physiological vulnerability that shapes post-fracture outcomes. The objective of this study is to evaluate osteoporosis-related care after fragility fracture and its association with frailty in adults hospitalized with fragility fractures in Riyadh, Saudi Arabia.

Methods

In this retrospective cohort study, electronic health records (EHRs) were reviewed for 301 adults admitted with fragility fractures at a tertiary hospital in Riyadh in 2024 (mean (SD) age, 72.8 (11.2) years; 52.2% women). Frailty was quantified using a cumulative deficit index built from 24 clinical, functional, and laboratory variables and categorized into quartile-based groups. Primary outcomes were prolonged hospitalization (>20 days) and mortality; secondary outcomes were unplanned readmission and refracture within one year.

Results

Anti-osteoporosis therapy was initiated during hospitalization in 8/301 patients (2.7%) and prescribed at discharge to 12/301 patients (4.0%); only 29 patients (9.6%) underwent follow-up bone mineral density (BMD) testing within one year. Out of 301 patients, 99 (32.9%) were classified as fit (frailty index (FI) ≤0.174), 63 (20.9%) as mildly frail, 67 (22.3%) as moderately frail, and 72 (23.9%) as severely frail. Compared with fit patients, patients with severe frailty had higher one-year mortality (19/72 (26.4%) vs 6/99 (6.1%); adjusted OR, 12.08; 95% CI, 1.54-94.89) and more prolonged hospitalizations (>20 days) (22/70 (31.4%) vs 2/90 (2.2%); adjusted OR, 6.38; 95% CI, 1.39-29.23). Associations with readmission and refracture were not statistically significant.

Conclusion

Osteoporosis care after fragility fracture was markedly underused, while a cumulative deficit FI identified patients at substantially higher risk of adverse outcomes. Routine frailty screening after fracture could help target secondary prevention efforts.

## Introduction

Fragility fractures are a major cause of morbidity and mortality and represent a growing burden on health care systems worldwide [1]. These fractures, typically resulting from low-energy trauma such as a fall from standing height, are increasingly recognized as sentinel clinical events reflecting underlying skeletal fragility rather than isolated orthopedic injuries [2]. Hip fractures, in particular, are associated with loss of independence, long-term disability, and excess mortality, with one-year mortality rates reported to reach 20% to 40% [3].

Osteoporosis, the underlying pathology in most fragility fractures, is a systemic skeletal disease characterized by low bone mass and deteriorated bone microarchitecture, which markedly increases fracture risk. Clinical guidelines recommend that patients presenting with fragility fractures should undergo assessment for osteoporosis and initiation of anti-osteoporotic therapy when indicated [4]. Despite these well-established recommendations, substantial gaps in care persist. Estimates vary by population and setting. International reports suggest that up to 80% of patients remain untreated after a fragility fracture, whereas a separate study of older women at high fracture risk in European primary care reported a treatment gap of 74.6% [5-7]. This deficiency contributes to recurrent fractures, functional decline, and increased health care utilization, despite evidence that timely therapy reduces the risk of subsequent fractures [8-10].

In clinical practice, patients with fragility fractures represent a clinically heterogeneous population with varying degrees of physiological reserve. Chronological age measures elapsed time and comorbidity count records diagnosed diseases, but neither fully captures multi-domain loss of reserve, functional dependence, cognition, nutrition, or vulnerability to an acute stressor. Patients of the same age and with similar comorbidity burdens may therefore have different postoperative risk. Frailty, conceptualized as the accumulation of health deficits, provides a framework for capturing this variability [11-14]. Frailty assessment may complement, rather than replace, age and comorbidity in post-fracture risk stratification; whether it adds measurable predictive performance requires formal model comparison.

Hospital admission for fracture offers a high-impact window to initiate secondary prevention. This includes ordering bone mineral density (BMD) testing, initiating pharmacotherapy, optimizing calcium and vitamin D status, and arranging specialist follow-up, ideally through multidisciplinary fracture liaison services that have demonstrated reductions in refracture and mortality [15,16]. However, most patients receive only surgical repair without secondary prevention, and recurrent fractures remain common, driving a cycle of morbidity, health care costs, and mortality.

Although frailty has been associated with adverse outcomes following orthopedic injury, most evidence derives from studies conducted outside the Middle East [17,18]. In Saudi Arabia, the burden of fragility fractures is expected to increase with population aging, yet data on post-fracture care and outcomes remain limited [19,20]. Accordingly, this study aimed to evaluate osteoporosis-related care following fragility fractures, quantify treatment gaps, and examine the association between frailty and clinically relevant outcomes in a tertiary hospital setting in Riyadh, Saudi Arabia.

## Materials and methods

Study design and setting

This retrospective observational cohort study was conducted using routinely collected data from the BestCare electronic health record (EHR) system at King Abdulaziz Medical City-Riyadh (KAMC-Riyadh), a tertiary academic hospital within the Ministry of National Guard Health Affairs (MNGHA) in Saudi Arabia. KAMC-Riyadh serves a defined beneficiary population, reducing cross-facility data fragmentation and ensuring comprehensive case capture. The study followed the Strengthening the Reporting of Observational Studies in Epidemiology (STROBE) guideline [21].

Study population

The study population consisted of all adults aged 50 years or older admitted with fragility fractures between the first of January and the end of December 2024. Fragility fractures were defined as fractures resulting from low-trauma mechanisms, such as a fall from standing height or less, or unspecified mechanisms without evidence of high-energy trauma. Patients were excluded if they sustained high-energy trauma (e.g., motor vehicle collisions, falls from greater than two meters, polytrauma, or open fractures from high-impact mechanisms). Additional exclusions included pathologic fractures secondary to malignancy or infection and fractures related to non-osteoporotic metabolic bone diseases such as osteogenesis imperfecta, Paget disease, untreated primary hyperparathyroidism, or osteomalacia. The age threshold of 50 years was selected to capture individuals at risk of age-related and postmenopausal osteoporosis, consistent with standard definitions of fragility fractures [22,23].

A non-probability consecutive sampling approach was used, whereby all eligible patients meeting the inclusion criteria during the study period were included. This cohort therefore represents a complete capture of fragility fracture admissions within the defined timeframe, minimizing selection bias within the source population.

Data sources and collection

Data were extracted from the integrated BestCare EHR system, which includes demographic information, comorbid conditions, ICD-10 diagnostic and procedural codes, admission and discharge records, operative reports, medication prescriptions, laboratory data, and imaging and BMD reports. Additional data on referrals and disposition outcomes were also retrieved. All eligible cases were identified through structured chart review. Extracted data were entered into a standardized database and underwent cleaning and validation procedures to ensure completeness and internal consistency prior to analysis.

Frailty index (FI) construction

A FI was constructed using the cumulative deficit model, following the approach described by Rockwood and Mitnitski [12,24]. Deficits were selected according to established criteria requiring that variables represent health-related conditions, increase in prevalence with age, span multiple physiological systems, and avoid early saturation within the study population [25,26]. The final FI incorporated 24 deficits across multiple domains based on the available variables in the EHR.

These included chronic comorbid conditions (diabetes mellitus, hypertension, cancer, cancer therapy, steroid use, and rheumatoid arthritis); acute clinical complications (delirium, pneumonia, urinary tract infection, thromboembolic events, acute coronary syndrome, heart failure, bleeding, malunion, and re-operation); functional impairments (requirement for assistance in daily activities and loss of independence); and fracture-related health indicators (previous fractures and multiple fractures in the same episode). Selected laboratory and physiological variables were incorporated after transformation using clinically relevant thresholds, including reduced estimated glomerular filtration rate (<60 mL/min/1.73 m²), abnormal adjusted calcium levels (<2.2 or >2.6 mmol/L), advanced age (categorized as <65 years "0", 65-79 years "0.5", and ≥80 years "1"), extremes of body weight (<60 kg or >100 kg), and menopause status as a marker of biological aging.

To avoid circularity and outcome leakage, variables that were also evaluated as outcomes (including post-fracture functional measures and in-hospital complications) were not included in the FI for the corresponding outcome models. For each outcome, an outcome-specific FI was recalculated by omitting that variable using a leave-one-out approach [27]. Each deficit was coded on a scale from 0 to 1, with binary variables assigned values of 0 (absence) or 1 (presence) and ordinal variables assigned intermediate values where appropriate [24]. To account for variable-specific missingness, the FI for each individual was calculated as the ratio of the number of deficits present to the number of non-missing deficits available for that individual, yielding a continuous score ranging from 0 to 1. For descriptive purposes, the index was categorized into quartile-based strata: fit (≤0.174), mild frailty (0.174-0.227), moderate frailty (0.227-0.318), and severe frailty (>0.318).

For descriptive and categorical analyses, frailty was classified using cohort-derived quartiles, an approach previously used in cumulative-deficit electronic frailty indices to provide interpretable strata when instrument-specific externally validated thresholds are unavailable; transferring fixed thresholds between frailty indices with different deficit compositions may not be appropriate without calibration.

Fracture risk assessment using the FRAX tool was not routinely documented in the EHR for this cohort. Although BMD testing was available for a subset of patients, incomplete data on key FRAX variables precluded retrospective calculation of FRAX scores. Accordingly, FRAX was not included in the analysis.

Outcomes and osteoporosis care measures

Primary outcomes included all-cause mortality during the index admission or within one-year follow-up and prolonged length of hospital stay, defined as greater than 20 days. Secondary outcomes included unplanned hospital readmission within one year, re-fracture at the same site within one year, and new fragility fracture at a different site within one year. In-hospital complications were also assessed, including delirium, pneumonia, urinary tract infection, thromboembolic events, clinically significant bleeding, malunion, and need for re-operation.

Patterns of osteoporosis-related care were evaluated, including vitamin D prescription during admission, anti-osteoporosis therapy initiation at different time points (pre-admission, during admission, at discharge, and outpatient post-discharge), referral to endocrinology, and BMD testing within one year.

Follow-up

Each patient was followed from the index admission date until the earliest of outcome occurrence, 12 months of follow-up, or the administrative censoring date. Because the study inclusion period extended through December 31, 2024, follow-up for outcomes continued beyond the inclusion window, with data extraction updated through December 31, 2025, to ensure complete one-year follow-up for all patients.

Ethical considerations

The study protocol was approved by the Institutional Review Board of the King Abdullah International Medical Research Center (KAIMRC). Given the retrospective nature of the study and the use of de-identified data, the requirement for informed consent was waived. All patient data were anonymized prior to analysis.

Statistical analysis

Continuous variables were summarized using means (SDs) and medians (IQRs), while categorical variables were presented as frequencies and percentages with 95% Wilson confidence intervals. Missing data were handled using variable-specific denominators.

The FI was analyzed as both a continuous and categorical variable using quartile-based cut-points to define four levels (fit to severely frail), with group comparisons conducted using Kruskal-Wallis tests for continuous variables and chi-square tests for categorical variables; Cramer's V was reported to quantify effect size. Associations between frailty and clinical outcomes (mortality, readmission, re-fracture, and prolonged hospitalization) were assessed using binary logistic regression models, with the fit group as the reference. To address sparse outcomes and reduce overfitting, L2-penalized logistic regression with ridge regularization was applied, and 95% confidence intervals were estimated using bootstrap resampling with 500 replications. Statistical significance was defined as a two-sided p-value <0.05, and analyses were conducted using R version 4.3.1 (R Foundation for Statistical Computing, Vienna, Austria).

Missing data

Missing data were reported for each variable, and analyses were conducted using available-case denominators without imputation.

## Results

Baseline characteristics, fracture profile, and initial bone health care

A total of 301 patients hospitalized with fragility fractures were included, with a mean (SD) age of 72.8 (11.2) years and a slight predominance of women (52.2%). The cohort carried a substantial comorbidity burden, most notably hypertension and diabetes mellitus, while rheumatoid arthritis was uncommon. Prior fragility fracture was reported in 17.3% of patients.

Hip fractures were the most frequent injury type, accounting for more than half of cases, followed by vertebral fractures. Most patients underwent surgical management, most commonly hemiarthroplasty or open reduction and internal fixation; 12.6% did not receive surgery. Median length of hospital stay was seven days (IQR, 5-12), with a wide range (0-251 days).

Markers of bone health management were notably suboptimal. Most serum calcium values were normal, though nearly one-quarter of patients had hypocalcemia. Vitamin D was deficient in 19.3% of patients, but values were missing for half the cohort. Renal function at admission was generally preserved (74.1% with eGFR ≥60 mL/min/1.73 m²); among patients with reduced eGFR, stage 2 was most common, followed by stage 3, while advanced disease (stages 4-5) was infrequent. Overall, the cohort reflects an older, multimorbid population with frequent calcium abnormalities, substantial missingness in vitamin D assessment, and limited uptake of osteoporosis-related preventive therapies (Table 1 and Figure 1).

**Table 1:** Baseline characteristics, clinical features, and care processes among patients hospitalized with fragility fractures (N = 301). All proportions were reported with 95% Wilson confidence intervals. Bolded rows spanning the n and % columns are section subheadings (e.g., CKD staging, fracture type); the merged cells are intentionally blank. The denominator is the full cohort, N = 301, unless otherwise noted.

Variable	n	% (95% Wilson CI)
Gender - Male	144	47.8 (42.3-53.5)
Gender - Female	157	52.2 (46.5-57.7)
Multiple fractures in same episode	39	13.0 (9.6-17.2)
CKD Staging (eGFR upon admission)		
Stage 1 (≥90)	151	50.2 (44.6-55.8)
Stage 2 (60-89)	72	23.9 (19.4-29.0)
Stage 3 (30-59)	53	17.6 (13.7-22.3)
Stage 4 (15-29)	10	3.3 (1.8-6.0)
Stage 5 (<15)	5	1.7 (0.7-3.8)
Bone Health Parameters (calcium levels)		
Adjusted serum calcium (n = 10 missing, 3.3%)		
Normal calcium	209	69.4 (64.0-74.4)
Hypocalcemia	72	23.9 (19.4-29.0)
Hypercalcemia	10	3.3 (1.8-6.0)
Vitamin D classification (n = 151 missing, 50.2%)		
Normal (50-125 nmol/L)	82	27.2 (22.5-32.5)
Deficient (<50 nmol/L)	58	19.3 (15.2-24.1)
High (>125 nmol/L)	10	3.3 (1.8-6.0)
Type of fracture		
Hip (most common site)	168	55.8 (50.2-61.3)
Vertebral	52	17.3 (13.4-22.0)
Wrist	34	11.3 (8.2-15.4)
Humerus	21	7.0 (4.6-10.4)
Ankle	13	4.3 (2.5-7.2)
Other	13	4.3 (2.5-7.2)
Comorbidities		
Diabetes mellitus	194	64.5 (58.9-69.6)
Hypertension	216	71.8 (66.4-76.5)
Active malignancy	41	13.6 (10.2-18.0)
Receiving chemotherapy	32	10.6 (7.6-14.6)
Chronic glucocorticoid use	23	7.6 (5.1-11.2)
Rheumatoid arthritis	6	2.0 (0.9-4.3)
Prior fragility fracture	52	17.3 (13.5-22.0)

**Figure 1:**
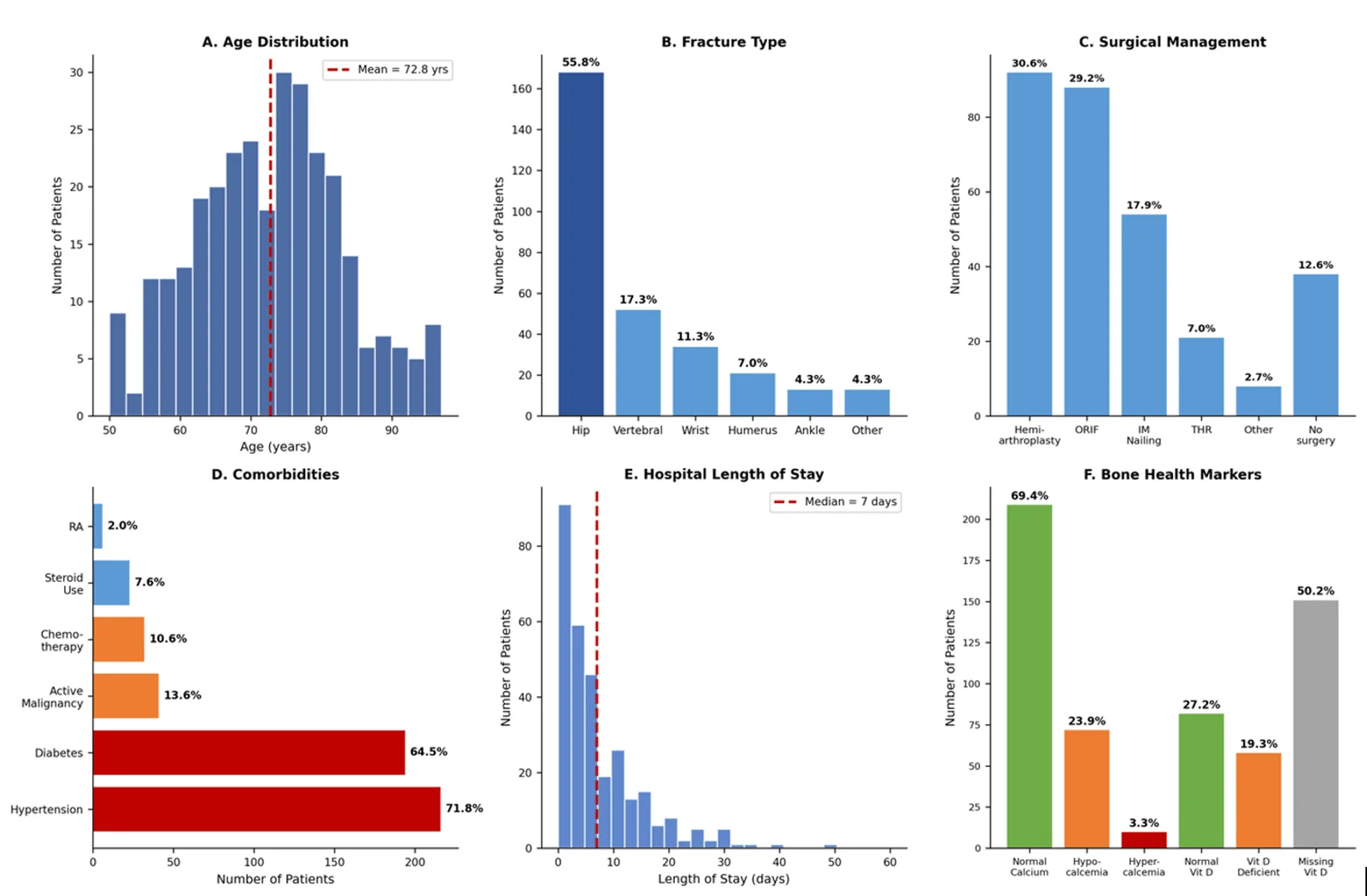
Baseline characteristics, clinical features, and care processes among patients hospitalized with fragility fractures (N = 301). Panel A shows the age distribution in the cohort. Panel B shows the prevalence of each fracture type. Panel C shows the percentage and type of surgeries performed. Panel D demonstrates the prevalence of each comorbid condition listed. Panel E shows the trend in length of stay (LOS). Panel F shows the remarks about bone health for these patients. ORIF: open reduction and internal fixation; IM nailing: intramedullary nailing; THR: total hip replacement; RA: rheumatoid arthritis

Osteoporosis care patterns, clinical outcomes, and post-fracture complications

Osteoporosis-related care was markedly limited: only 4.7% of patients were receiving anti-osteoporosis therapy before admission, 2.7% during hospitalization, and 4.0% at discharge. Follow-up bone density testing within one year occurred in only 9.6% of patients, and only 6.3% received an inpatient endocrinology consultation despite referral in 28.9%.

At one-year follow-up, all-cause mortality occurred in 10.6% of patients and unplanned readmission in 7.3%. Refracture at the same site occurred in 10.3% of patients; new fragility fracture at a different site was less frequent (2.0%).

Functional outcomes reflected a high burden of post-fracture disability: 56.4% of patients required assistance with daily activities and 30.6% experienced loss of independence. Urinary tract infection was the most common in-hospital complication, followed by delirium and pneumonia (Table 2).

**Table 2:** Management, osteoporosis care, clinical outcomes, and complications among patients hospitalized with fragility fractures (N = 301). All proportions are reported with 95% Wilson confidence intervals. Bolded rows spanning the n, %, and Notes columns are section subheadings (e.g., anti-osteoporosis therapy, clinical outcomes); the merged cells are intentionally blank. A dash (-) in the Notes column indicates no additional note. BMD: bone mineral density; DVT: deep vein thrombosis; ORIF: open reduction and internal fixation; PE: pulmonary embolism

Variable/Response	n	% (95% Wilson CI)	Notes
Surgery required	263	87.4 (83.1-90.7)	-
Surgical management			
Hemiarthroplasty	92	30.6 (25.6-36.0)	-
ORIF	88	29.2 (24.4-34.6)	-
Intramedullary nailing	54	17.9 (14.0-22.7)	-
Total hip replacement	21	7.0 (4.6-10.4)	-
Other	8	2.7 (1.4-5.2)	-
No surgery	38	12.6 (9.3-16.9)	-
Referred to endocrinologist (for any indication)	87	28.9 (24.1-34.3)	-
Endocrinology consultation (inpatient)	19	6.3 (4.1-9.6)	-
Vitamin D prescribed during admission	36	12.0 (8.8-16.2)	-
Anti-osteoporosis therapy			
Pre-admission (Pre-existing treatment)	14	4.7 (2.8-7.7)	-
During admission (Initiated inpatient)	8	2.7 (1.4-5.2)	-
Prescribed at discharge	12	4.0 (2.3-6.9)	-
Outpatient (deferred post-discharge)	40	13.3 (9.9-17.6)	-
Follow-up BMD within 1 year	29	9.6 (6.8-13.5)	-
Clinical outcomes (one-year follow-up)			
All-cause mortality	32	10.6 (7.6-14.6)	-
Unplanned readmission	22	7.3 (4.9-10.8)	-
Refracture at same site	31	10.3 (7.4-14.2)	-
New fragility fracture at different site	6	2.0 (0.9-4.3)	Small event count
Functional outcomes			
Requires assistance in daily activities (N = 289; 12 missing)	163	56.4 (50.6-62.0)	-
Loss of independence (N = 288; 13 missing)	88	30.6 (25.5-36.1)	-
In-hospital complications			
DVT/PE	16	5.5 (3.4-8.7)	N=292
Delirium	23	7.8 (5.3-11.5)	N=293
Clinically significant bleeding	2	0.7 (0.2-2.5)	N=291; near-zero variance
Pneumonia	18	6.0 (3.8-9.3)	-
Urinary tract infection	33	11.0 (7.9-15.0)	-
Malunion	5	2.0 (0.8-4.5)	N=255; 46 missing
Re-operation required	13	4.5 (2.6-7.5)	N=290

Frailty distribution and association with clinical outcomes

Among 301 patients, 99 (32.9%) were classified as fit (FI ≤ 0.174), representing the lowest cohort-derived FI category; 63 (20.9%) were mildly frail, 67 (22.3%) were moderately frail, and 72 (23.9%) were severely frail. FI scores increased stepwise across categories, confirming appropriate stratification (p < 0.001), and increasing frailty was associated with older age (p = 0.001) and, without a consistent gradient, a higher proportion of female patients (ranging from 42.4% in fit to 65.7% in moderate frailty; p = 0.013) (Table 3 and Figure 2).

**Table 3:** Frailty index and demographic characteristics, and clinical outcomes (univariable and multivariable-adjusted analysis), by cumulative deficit frailty index category (N = 301). FI v2 (24 deficits). Frailty categories were defined using data-driven quartile cut-points: fit (≤0.174), mild frailty (0.174-0.227), moderate frailty (0.227-0.318), and severe frailty (>0.318). Odds ratios were adjusted for age (continuous) and sex and estimated using L2-penalized (ridge) logistic regression; 95% CIs were derived from 500 bootstrap replications. Reference category: fit. Nagelkerke R²: mortality, 0.16; readmission, 0.08; refracture, 0.04; prolonged LOS, 0.22. N/A indicates a row for which an adjusted odds ratio was not estimated (descriptive characteristic, not a modeled outcome). ^a^ Adjusted odds ratios compare each frailty category with the fit (reference) category. *p<0.05 vs fit. aOR: adjusted odds ratio; CI: confidence interval; LOS: length of stay

Variable/Outcome	Fit (n=99)	Mild frailty (n=63)	Moderate frailty (n=67)	Severe frailty (n=72)	Univariable p (Cramer's V)	Mild frailty aOR (95% CI)^a^	Moderate frailty aOR (95% CI)^a^	Severe frailty aOR (95% CI)^a^
Frailty index and demographic characteristics								
FI score, mean (SD)	0.133 (0.039)	0.214 (0.013)	0.279 (0.022)	0.405 (0.063)	<0.001	N/A	N/A	N/A
Age, y, mean (SD)	69.8 (12.9)	72.1 (10.0)	74.2 (11.3)	76.0 (8.2)	0.001	N/A	N/A	N/A
Female sex, n (%)	42 (42.4)	29 (46.0)	44 (65.7)	42 (58.3)	0.013	N/A	N/A	N/A
LOS, d, median (IQR)	6 (4-8)	8 (5-11)	8 (5-12)	12 (7-23)	<0.001	N/A	N/A	N/A
Clinical outcomes, n/N (%)								
									Mortality (1-y)	6/99 (6.1)	2/63 (3.2)	5/67 (7.5)	19/72 (26.4)	<0.001 (V=0.290)	2.91 (0.35-24.00)	4.24 (0.50-35.76)	12.08 (1.54-94.89)*
Unplanned readmission (1-y)	3/99 (3.0)	0/63 (0.0)	9/67 (13.4)	10/72 (13.9)	0.001 (V=0.230)	0.17 (0.01-1.89)	2.07 (0.41-10.46)	4.11 (0.87-19.37)
Refracture, same site (1-y)	8/99 (8.1)	3/63 (4.8)	8/67 (11.9)	12/72 (16.7)	0.113 (V=0.141)	0.80 (0.20-3.25)	1.78 (0.45-6.99)	2.67 (0.71-10.08)
Prolonged LOS, >20 d	2/90 (2.2)	5/59 (8.5)	3/63 (4.8)	22/70 (31.4)	<0.001 (V=0.260)	1.42 (0.29-6.97)	1.43 (0.27-7.74)	6.38 (1.39-29.23)*

**Figure 2:**
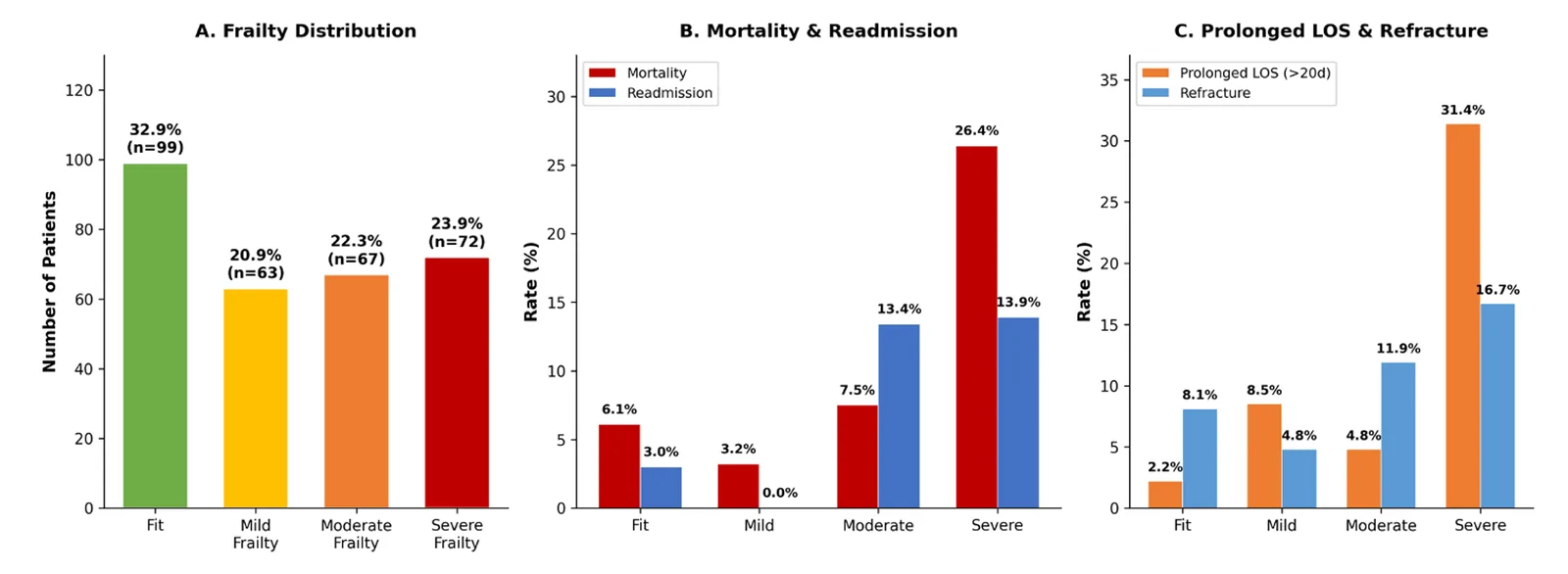
Frailty distribution and clinical outcomes across frailty categories (N = 301). Panel A shows frailty distribution by quartile-based frailty index (FI) strata. Panel B shows mortality and readmission rates. Panel C shows prolonged length of stay (LOS) and refracture rates.

Frailty was associated with adverse clinical outcomes. Mortality increased markedly across frailty categories, from 6.1% in fit patients to 26.4% in those with severe frailty (p < 0.001). Hospital readmission was also higher with increasing frailty (3.0% in fit vs 13.9% in severe frailty; p = 0.001), while refracture at the same site showed a non-significant trend (8.1% vs 16.7%; p = 0.113). Length of stay increased with frailty severity, from a median of 6 (4-8) days in fit patients to 12 (7-23) days in severe frailty (p < 0.001), and prolonged hospitalization (>20 days) occurred in 31.4% of severely frail patients versus 2.2% of fit patients (p < 0.001) (Table 3).

Multivariable analysis

In multivariable analyses adjusted for age and sex, increasing frailty was independently associated with higher odds of mortality and prolonged hospitalization, while associations with readmission and refracture were not statistically significant (Table 3). Compared with fit patients, those with severe frailty had higher one-year mortality (19/72 (26.4%) vs 6/99 (6.1%); adjusted OR, 12.08; 95% CI, 1.54-94.89; p = 0.02) and more prolonged hospitalizations of more than 20 days (22/70 (31.4%) vs 2/90 (2.2%); adjusted OR, 6.38; 95% CI, 1.39-29.23; p = 0.02).

Risk of unplanned readmission and refracture also trended upward with greater frailty severity but did not reach statistical significance (severe frailty: readmission OR, 4.11; 95% CI, 0.87-19.37; refracture OR, 2.67; 95% CI, 0.71-10.08). Model performance was modest, with Nagelkerke R² values of 0.16 for mortality and 0.22 for prolonged length of stay (Table 3 and Figure 3).

**Figure 3:**
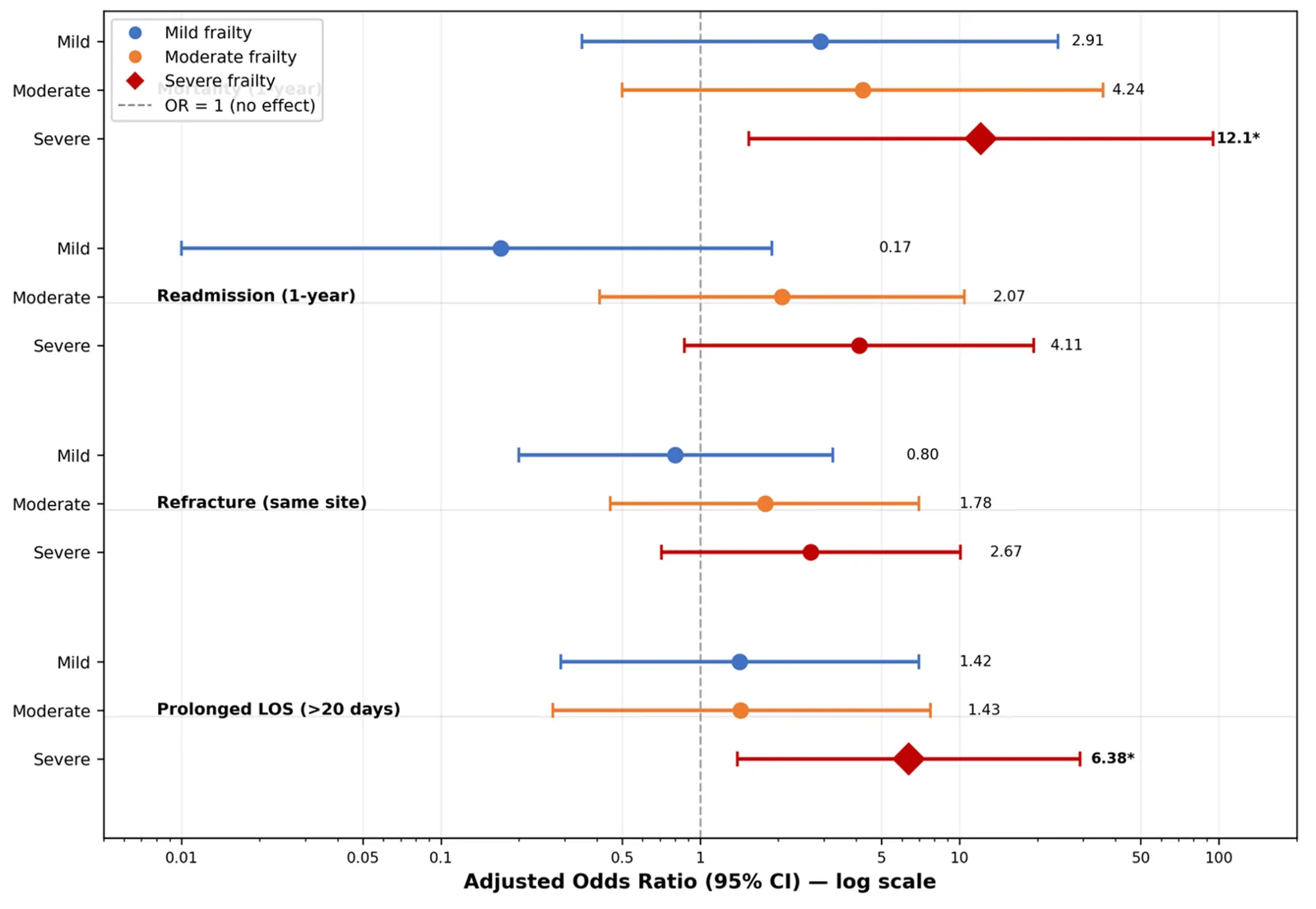
Association between frailty category and clinical outcomes. Adjusted odds ratios (95% CI) from L2-penalized logistic regression (ridge), adjusted for age and sex. Reference category: fit. Blue = mild, orange = moderate, red = severe frailty. Diamond markers indicate p < 0.05.

## Discussion

In this cohort of adults hospitalized with fragility fractures, we observed three principal findings. There were persistent gaps in osteoporosis-related care following the fracture episode, a high burden of adverse clinical and functional outcomes, and, finally, an association between frailty and worse outcomes that may inform clinical risk assessment, though further validation is needed before predictive use.

Osteoporosis-related care was markedly suboptimal across all stages. Despite fragility fractures being well-established sentinel events for osteoporosis, fewer than 5% of patients received anti-osteoporosis therapy during admission or at discharge, and less than 10% underwent follow-up BMD testing. These findings align with international literature showing most fragility fracture patients do not receive appropriate osteoporosis evaluation or treatment, with care gaps exceeding 70% in many settings [5,6]. Similar deficiencies have been reported in Saudi Arabia, where national reports emphasize persistent under-diagnosis, limited BMD access, and low post-fracture treatment rates [19,20]. The low rates of endocrinology referral and inpatient consultation in our cohort further underscore missed opportunities for coordinated post-fracture care, including fracture liaison services, which improve treatment uptake and reduce subsequent fracture risk [15,16].

The fragility fracture population was characterized by advanced age, high comorbidity burden, and predominance of hip fractures - all known predictors of poor outcomes. High rates of hypertension and diabetes reflected the broader epidemiologic profile of aging populations in the region. Prior regional and international studies report one-year mortality of 20%-30% after hip fracture [28], whereas our 10.6% mortality likely reflects a broader range of fracture types. Still, the high rate of functional decline, with more than half of patients requiring assistance with daily activities, highlights the substantial functional burden associated with fragility fractures.

Frailty, measured by a cumulative deficit index tailored to this population, showed a clear, clinically meaningful gradient across outcomes. Severely frail patients had markedly higher mortality and longer hospital stays, consistent with frailty's established role as a predictor of adverse outcomes in hospitalized and surgical populations [14]. Unlike many prior studies using generic frailty measures, our index incorporated deficits specific to fragility fracture patients, including comorbidities, functional impairment, and fracture-related complications. Frailty was less strongly associated with readmission and refracture, possibly reflecting the multifactorial nature of these events, including post-discharge care, rehabilitation, and treatment adherence. Wide confidence intervals for these secondary outcomes suggest the analyses were underpowered, and non-significance should not be interpreted as evidence of no association.

A particularly important finding is the lack of alignment between patient risk and care delivery. Patients with higher frailty, those at greatest risk of mortality and prolonged hospitalization, were not more likely to receive osteoporosis evaluation or treatment. This mismatch suggests current care pathways do not adequately incorporate risk stratification and may fail to prioritize those most likely to benefit from intervention. Given that fragility fractures are preventable and effective therapies exist to reduce subsequent fracture risk, this represents a major opportunity for improving outcomes.

This study has important clinical implications. Fragility fractures should be consistently recognized as a trigger for comprehensive osteoporosis management. Validated instruments such as the nine-point Clinical Frailty Scale and the 36-deficit electronic FI are available and should be preferred for routine clinical screening when their required data can be collected reliably [29,30]. Other researched tools include the modified FI, Hospital Frailty Risk Score, FRAIL scale, Nottingham Hip Fracture Score, and Hip-Multidimensional Frailty Score; FRAX estimates future fracture probability but does not measure frailty. The locally constructed index in this study was used to examine association, not to create a bedside prediction rule. Developing a new fracture-specific score would require a separate, adequately powered study with pre-specified predictors, internal resampling, independent external evaluation, calibration assessment, and comparison with existing instruments. System-level strategies, including fracture liaison services and standardized care pathways, remain necessary to close the osteoporosis care gap.

Limitations

This study has several limitations. First, its retrospective design using EHR data may be subject to misclassification and incomplete capture of clinical variables, particularly outcomes and care occurring outside the study institution. Second, the FI's deficits were limited to variables available in the dataset and may not fully capture dimensions such as cognitive function, social support, or detailed functional assessments. Third, missing data for certain variables may have introduced bias despite reporting denominators for non-missing values. Fourth, the small number of events for some outcomes, particularly refracture and complications, resulted in wide confidence intervals and limited statistical power. Fifth, the study was conducted at a single tertiary center in Saudi Arabia, limiting the generalizability of the findings. Furthermore, the proposed FI was developed and evaluated within this cohort and has not yet undergone external validation. Therefore, its performance and applicability in other healthcare settings, populations, and geographic regions remain uncertain and should be confirmed in independent multicenter cohorts before broader clinical implementation. Sixth, information on post-discharge treatment adherence and outpatient follow-up was limited. Finally, although vitamin D deficiency was evaluated as a study variable, vitamin D measurements were unavailable for a proportion of participants. This may have introduced selection bias and limited the interpretation and generalizability of vitamin D-related findings. However, because vitamin D was not included in the construction of the FI or the primary multivariable regression models, the missing data are unlikely to have materially influenced the principal study findings.

## Conclusions

In this cohort of adults hospitalized with fragility fractures, osteoporosis care was markedly suboptimal despite substantial morbidity and mortality. A cumulative deficit FI provided clinically meaningful risk stratification, with higher frailty being associated with increased risk of mortality and prolonged hospitalization. These findings highlight a critical mismatch between patient risk and care delivery and support integrating frailty assessment into post-fracture care pathways to improve secondary fracture prevention and clinical outcomes.
